# Median Arcuate Ligament Syndrome: A Disease of Mind, Body, or Both?

**DOI:** 10.7759/cureus.110779

**Published:** 2026-06-13

**Authors:** Sai Sravani Sure, Narayanan Cunnigaiper, Lakshmidharan Mohan, Radhakrishnan Raju

**Affiliations:** 1 Department of Medicine, Sri Ramachandra Institute of Higher Education and Research, Chennai, IND; 2 Department of General Surgery, Sri Ramachandra Institute of Higher Education and Research, Chennai, IND; 3 Department of Vascular Surgery, Sri Ramachandra Institute of Higher Education and Research, Chennai, IND

**Keywords:** celiac artery, gastrointestinal quality of life index, kessler-10 score, laparoscopic decompression, median arcuate ligament, psychological distress

## Abstract

Background

Median arcuate ligament syndrome (MALS) is a rare condition resulting from an anatomical variation in the placement of the median arcuate ligament, which compresses the celiac artery. The difficulty in diagnosing this condition may result in chronic underdiagnosis and associated psychological distress in patients.

Methods

This is a single-center retrospective review of five patients diagnosed with MALS who underwent laparoscopic celiac artery decompression performed by a single senior consultant surgeon between April 2020 and April 2025. Institutional Ethics Committee approval (CSP-III/25/OCT/27/405) and consent were obtained from all participants. The primary outcome measure of the study was to evaluate subjective improvement along with gastrointestinal and vascular parameters following successful laparoscopic decompression of the celiac artery. Preoperative psychological assessment was not performed, and scores were obtained using a recall-based method. The Gastrointestinal Quality of Life Index (GIQLI) and the Kessler Psychological Distress Scale (K10) were used to assess gastrointestinal symptoms and psychological condition of the patients pre- and postoperatively, and were compared for each patient.

Results

All patients demonstrated significant improvement in gastrointestinal symptoms (p=0.043) and normalization of vascular parameters following laparoscopic decompression of the celiac artery. Psychological scores improved (p=0.042); however, complete subjective improvement was not observed in all patients, as postoperative evaluation revealed findings suggestive of generalized anxiety disorder in all patients, with two patients reporting depression.

Conclusion

In this study, laparoscopic decompression of the celiac artery did not result in complete subjective improvement, despite successful anatomical correction. Despite the small sample size, these findings emphasize the need for a standardized criterion and scoring system to guide surgical decision-making in MALS, given its complex, multifactorial nature.

## Introduction

Median arcuate ligament syndrome (MALS) is a rare vascular condition caused by compression of the celiac artery by the median arcuate ligament in a few individuals, either due to a low-lying ligament or a highly placed celiac artery [1]. The estimated incidence of the condition is approximately two per 100,000 population [2,3]. However, radiological celiac artery compression may be observed in up to 10-24% of individuals, often as an incidental finding [3].

Despite effective restoration of vascular parameters following successful laparoscopic decompression of the celiac artery, complete subjective improvement could not be appreciated in the patients. Several studies have been conducted to demonstrate the prevalence of psychological comorbidities in patients with MALS and have proven that a reduction in abdominal pain does not necessarily eliminate psychological distress [4-6]. We aimed to better understand the persistence of psychological symptoms following successful laparoscopic decompression. We employed the (1) Gastrointestinal Quality of Life Index (GIQLI) to assess the improvement in gastrointestinal (GI) symptoms [7], (2) Kessler Psychological Distress Scale (K10) to assess improvement in psychological symptoms [8], and (3) Doppler ultrasonography (DUS) to assess vascular parameters. Using these tools, we aimed to evaluate the hypothesis that psychological recovery does not necessarily parallel vascular or gastrointestinal improvements following surgery.

## Materials and methods

Study design and setting

This is a single-center retrospective review of five consecutive patients with MALS who underwent laparoscopic celiac artery decompression between April 2020 and April 2025 in the General Surgery Department of a teaching hospital. Institutional Ethics Committee approval (CSP-III/25/OCT/27/405) was obtained, and consent was taken from all participants. 

Participants and data collection

**Figure 1:**
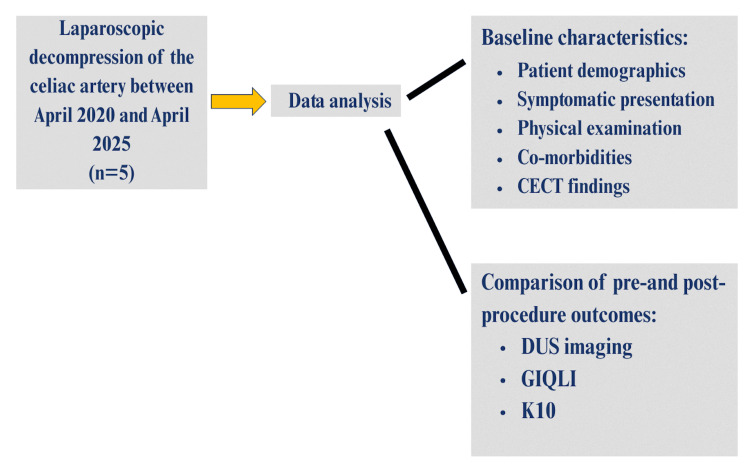
Flow chart depicting study design. There was no loss to follow-up. CECT: contrast-enhanced computed tomography; DUS: Doppler ultrasonography; GIQLI: Gastrointestinal Quality of Life Index; K10: Kessler Psychological Distress Scale.

All the participants were operated on by a single senior consultant surgeon with over 28 years of experience. Patients ≤18 years were excluded. Pre- and intra-operative data were collected retrospectively from the hospital medical records. Gastrointestinal and psychological outcomes were assessed using GIQLI and K10 questionnaires. Each questionnaire was administered twice by the principal investigator, with preoperative scores obtained using a recall-based method and postoperative scores obtained during the follow-up period. Pre- and postoperative scores were compared. A flow chart summarizing the study design, patient selection, and outcome assessment is shown in Figure 1.

Intervention

**Figure 2:**
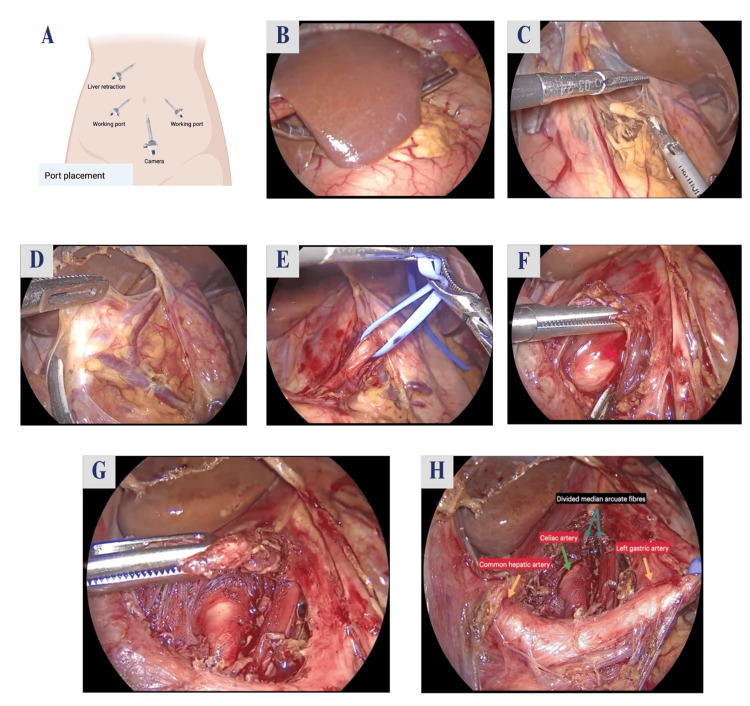
Intra-operative images depicting laparoscopic decompression of the celiac artery. (A) Placement of ports, (B) retraction of liver, (C) entering the pars flaccida, (D) isolating the LGA, (E) LGA looped and retracted, (F) median arcuate fibers divided, (G) complete circumferential release of the vessel achieved, and (H) completion image showing the surrounding structures. LGA: left gastric artery. Figure part label (A) was created by the authors using the BioRender platform (BioRender.com Inc., Toronto, ON, Canada).

Laparoscopic decompression of the celiac artery was performed as depicted in Figure 2. Four ports were placed, along with an additional port for retraction of the stomach, esophagus, or liver (Figures 2A, 2B). The celiac artery was approached via the pars flaccida (Figure 2C). The left gastric artery (LGA) was exposed, looped, and retracted (Figures 2D, 2E). The thick constricting muscle and nerve fibers around the celiac artery were divided using harmonic shears (Figure 2F). Complete circumferential release of the celiac artery was performed, and the left gastric, common hepatic, and splenic arteries were devoid of fibers (Figures 2G, 2H). Intra-operative DUS confirmed the adequacy of decompression. Loss of respiratory variation in PSV with the Valsalva maneuver was considered the endpoint of adequate decompression. 

The operative steps are demonstrated in Video 1.

**Video 1:** Operative video-laparoscopic release of the median arcuate ligament.

Follow-up and outcomes

All patients had a minimum follow-up of six months, with no loss to follow-up. Clinical evaluation and DUS examination were performed. Patients received paracetamol for post-operative pain, with no requirement for long-term analgesics. 

Data analysis

The data collected were entered into Microsoft Excel 2016 (Microsoft Corporation, Redmond, Washington, USA) and analyzed using IBM SPSS Statistics for Windows, Version 29.0 (IBM Corp, Armonk, NY). Descriptive statistics (mean, median, IQR, and SD) were used for continuous variables. The Wilcoxon signed-rank test was used to find the significant difference between the bivariate samples in paired groups. In the above statistical tool, a probability of less than 0.05 was considered significant, which was indicated by an asterisk (*) along with p-values in the text and tables.

## Results

Baseline characteristics of the study participants

Five participants (three females and two males) who underwent laparoscopic decompression of the celiac artery for MALS were included in the study. The mean age of the participants was 46.8 years, with a mean body mass index of 21.8 kg/m^2^. Four out of five patients reported weight loss, with a mean weight loss of 5.4 kg over an average duration of 19 months. All participants had post-prandial epigastric pain and loss of appetite in common. Associated gastrointestinal symptoms such as early satiety (n=5, 100%) and bloating (n=4, 80%) were more commonly reported than nausea and vomiting (n=2, 40%). Comorbidities included dyslipidemia (n=5, 100%), diabetes mellitus (n=4, 80%), and systemic hypertension (n=3, 60%), with two patients (40%) having a history of smoking. The mean duration of symptoms before the first presentation to our department was 3.8 ± 1.4 years.

**Figure 3:**
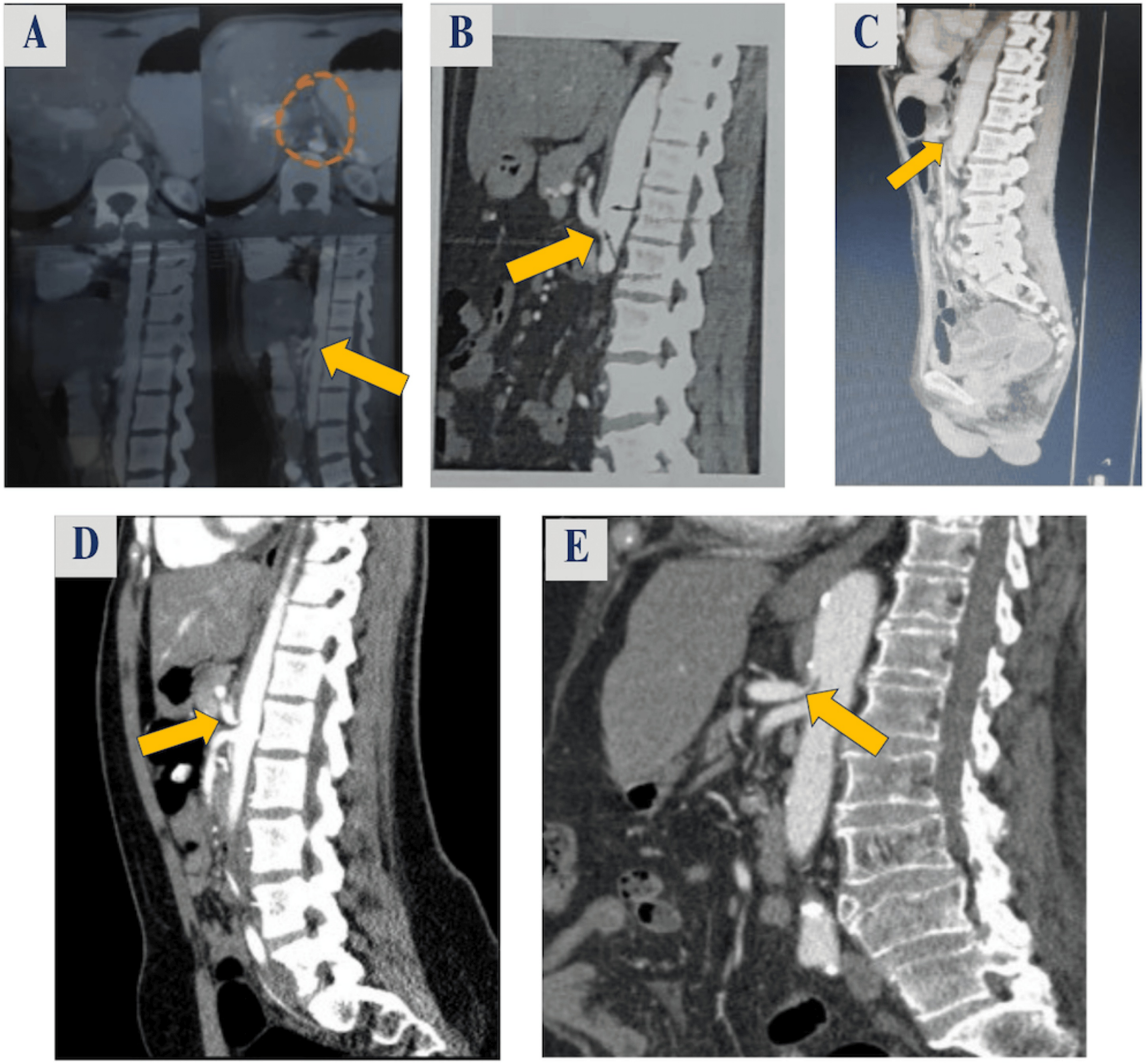
Pre-operative CECT abdomen images. The figure depicts pre-operative CECT images of five patients (A)-(E) showing stenosis of the celiac artery with post-stenotic dilatation (indicated by the yellow arrows). CECT: contrast-enhanced computed tomography.

All patients underwent contrast-enhanced computed tomography (CECT) prior to the laparoscopic procedure, as depicted in Figure 3.

Pre-operative CECT demonstrated significant proximal celiac artery compression (60-70%) in all patients (Figure 3). The length of stenosis ranged from 5 to 8.5 mm, with post-stenotic dilatation noted in all patients.

Pre- and postoperative DUS examinations were successfully performed in all patients, with no loss to follow-up. The findings were noted and compared (Table 1).

**Table 1:** Comparison of pre- and postoperative DUS findings. PSV: peak systolic velocity, EDV: end diastolic velocity, DUS: Doppler ultrasonography. *Refers to statistically significant value (p<0.05).

DUS parameters	Pre-op values	Post-op values	p-value
	Mean ± SD (median (Q1-Q3))	Mean ± SD (median (Q1-Q3))	
Celiac artery PSV during expiration (cm/s)	320 ± 31.6 320 (290-350)	146 ± 9.6 145 (137.5-155)	0.043*
Celiac artery PSV during inspiration (cm/s)	191 ± 14.3 190 (177.5-205)	139 ± 7.4 140 (132.5-145)	0.042*
Celiac to aortic PSV ratio	3.8 ± 0.3 3.8 (3.5-4.1)	1.4 ± 0.1 1.4 (1.4-1.6)	0.043*
EDV (cm/s)	61.6 ± 5.9 60 (56.5-67.5)	28.6 ± 2.4 28 (26.5-31)	0.042*

DUS evaluation showed a significant postoperative reduction in celiac artery PSV during expiration (0.043*) and inspiration (0.042*), celiac to aortic PSV ratio (0.043*), and EDV (0.042*). Resolution of post-stenotic turbulence was noted in all patients following the procedure.

The mean operating time was 110 ± 32 minutes with no intra- or postoperative complications. None of the patients required endovascular interventions or revision surgery, as there was significant improvement in hemodynamic parameters on DUS following ligament release. Celiac ganglionectomy was not performed in any patients. A mean weight gain of 6.2 ± 2.4 kg over an average duration of 12 months was noted.

Preoperative GIQLI and K10 scores were obtained using the recall method and were compared against postoperative scores (Table 2). Postoperative assessment revealed findings suggestive of generalized anxiety disorder in all patients, with two patients reporting depression.

**Table 2:** Comparison of pre-and post-operative GIQLI and K10 scores among study participants. *Refers to statistically significant value (p<0.05). GIQLI: Gastrointestinal Quality of Life Index; K10: Kessler Psychological Distress Scale.

Parameter	Pre-op values	Post-op values	p-value
	Mean ± SD (median (Q1-Q3))	Mean ± SD (median (Q1-Q3))	
GIQLI score	72.4 ± 9.4; 72 (64-81)	127 ± 4.7; 128 (122.5-131)	0.043*
K10 score	37.4 ± 6; 38 (31.5-43)	28.2 ± 4.6; 28 (24-32.5)	0.042*

GIQLI scores improved significantly following surgery (p=0.043*). Although a significant improvement was noted in K10 scores (p=0.042*), complete normalization of the symptoms was not observed (Table 2). 

## Discussion

The current study showed that laparoscopic decompression of the celiac artery significantly improved both hemodynamic parameters and gastrointestinal symptoms (p=0.043*) in all patients. There was a marked reduction in PSV during both expiration (p=0.043*) and inspiration (p=0.042*), along with improvement in EDV (p=0.042*) and celiac to aortic PSV ratio (p=0.043), indicating effective restoration of celiac artery blood flow.

Similar studies have been conducted among patients with MALS. A large cross-sectional study conducted among 763 patients (578 open cases and 185 laparoscopic cases) showed lower rates of hospital stay, complications, reoperation, and mortality in the laparoscopic group compared with the open group, showing the efficacy of the laparoscopic procedure in treating the condition [9]. A single-center prospective study reported effective symptomatic resolution in 11 patients who underwent laparoscopic decompression of the celiac artery [10]. Early return to work and effective pain relief were reported in a patient who underwent laparoscopic decompression of the celiac artery [11].

However, the patient reported that surgical outcomes are not always as expected and depend on multiple patient and disease-related factors. The absence of a standardized scoring system makes the diagnosis of MALS challenging. Currently, the diagnosis relies on a combination of clinical features suggestive of chronic mesenteric ischemia (CMI) and compression of the celiac artery on CECT. A PSV >200 cm/s during deep expiration, with a drop in PSV during inspiration, along with a dynamic variation (DPSV) of >60 cm/s, is necessary to diagnose the condition. An EDV >55 cm/s during expiration indicates hemodynamically significant stenosis. A celiac to aortic PSV ratio of >3:1 is considered to be significant. Endovascular intervention (angiography with angioplasty) is indicated in a case of flow-reducing stenosis of >70%. Celiac ganglionectomy with greater splanchnic nerve block is performed in patients with persistent postprandial abdominal pain eight weeks after surgery, with normal hemodynamic parameters on DUS and normal computed tomography angiography (CTA) [4,12].

Although laparoscopy proves to be efficacious for anatomical correction of the condition, complete subjective improvement was not achieved in the patients. K10 scores improved significantly (p=0.042*) after surgery, but none of the patients achieved complete normalization of psychological scores. This suggests that psychological improvement may not always parallel surgical correction in all patients; however, given the small sample size, these findings should be interpreted cautiously. These results align with the existing literature that suggests laparoscopic decompression can only correct the mechanical component of MALS but not the autonomic dysregulation, thereby resulting in incomplete subjective satisfaction [13-15].

Strengths of the study

All patients included in the study were consecutive cases operated on by a single senior consultant surgeon, thereby avoiding inter-observer variation. The simultaneous use of two validated questionnaires to assess GI and psychological symptoms, along with CECT and DUS, helped to strengthen the findings. 

Limitations of the study

This is a single-center study with a small sample size; therefore, the p-values should be interpreted cautiously. The absence of a comparator group limits the assessment of the relative efficacy of the procedure. The retrospective collection of preoperative psychological and gastrointestinal scores using patient recall may introduce substantial recall bias.

Recommendations for future research

Future studies with larger sample sizes and long-term follow-ups, involving comparator groups with a multidisciplinary approach, are recommended to establish a standardized diagnostic and scoring system for effective clinical decision-making. Postoperative CECT can be performed to evaluate and compare the anatomical improvement.

## Conclusions

MALS is a rare, often under-recognized condition with variable clinical presentations. While it may be identified incidentally on imaging, a subset of patients experiences significant symptoms requiring intervention. While anatomically correcting the constricting ring around the celiac axis, we did not observe complete subjective improvement, and the patients' clinical condition remained unchanged despite weight gain. Although statistically significant improvements were observed, the small sample size limits generalizability. It is important to remember that, like a reflux or anti-reflux procedure, definitive guidance should be available to determine the necessity of surgery in these patients, and a scoring system should be developed.
